# Efficacy of computed tomography in diagnosing pulmonary hypertension: A systematic review and meta-analysis

**DOI:** 10.3389/fcvm.2022.966257

**Published:** 2022-10-05

**Authors:** Riken Chen, Huizhao Liao, Zhenan Deng, Zhenfeng He, Zhenzhen Zheng, Jianmin Lu, Mei Jiang, Xiaofeng Wu, Wenliang Guo, Zijie Huang, Huimin Chen, Cheng Hong, Nanshan Zhong

**Affiliations:** ^1^State Key Laboratory of Respiratory Disease, National Clinical Research Center for Respiratory Disease, Guangzhou Institute of Respiratory Health, The First Affiliated Hospital of Guangzhou Medical University, Guangzhou, Guangdong, China; ^2^Department of Respiratory and Critical Care Medicine, The Second Affiliated Hospital of Guangdong Medical University, Zhanjiang, Guangdong, China; ^3^Department of Traditional Chinese Medicine, The Second Affiliated Hospital of Guangdong Medical University, Zhanjiang, Guangdong, China

**Keywords:** pulmonary hypertension, meta-analysis, diagnostic, computed tomography, X-ray

## Abstract

**Objective:**

This study seeks to evaluate the diagnostic value of computed tomography (CT) in pulmonary hypertension.

**Method:**

PubMed, Embase, Scopus, and Web of Science databases were searched to obtain the relevant English literature, and the retrieval time until June 2022. The quality of the included studies is evaluated using the QUADAS-2 tool. The quality of the included studies was assessed, followed by a meta-analysis, analyze heterogeneity, summarize sensitivity and specificity, draw the comprehensive subject working characteristics (sROC) curve, calculate the area under the curve and conduct subgroup analysis and sensitivity analysis to find the source of the heterogeneity.

**Results:**

A total of 12 articles were included, all with pulmonary artery diameter/liter aortic diameter >1 or 1 as the diagnostic criteria for pulmonary hypertension, and a total of 1,959 patients were included. Deek’s funnel plot analysis suggests that there is no significant publication bias (*P* = 0.102). The combined sensitivity was 0.652 (95% CI: 0.579, 0.719), combined specificity was 0.830 (95% CI: 0.796, 0.880), positive likelihood ratio was 3.837 (95% CI: 3.215, 4.579), negative likelihood ratio was 0.419 (95% CI: 0.346, 0.507), diagnostic odds ratio was 9.157 (95% CI: 6.748, 12.427) and area under the summary receiver operating characteristic (SROC) curve was 0.84 (95% CI: 0.81, 0.87).

**Conclusion:**

The CT examination of pulmonary artery diameter/aortic artery hypertension is worthy of clinical application.

## Introduction

Pulmonary hypertension (PH) is a pathophysiological state of abnormal elevated pulmonary artery pressure due to multiple known or unknown causes, and its diagnostic criteria are sea level, resting state and 25 mmHg mean pulmonary artery pressure (mPAP) measured with a right heart catheter (1, 2). It is a fatal disease with poor long-term prognosis and high mortality, and common pathophysiological and histological features of pulmonary vasoconstriction, small pulmonary artery reconstruction, and thrombosis (3, 4). These pathological changes lead to increased pulmonary vascular resistance that eventually causes right ventricular (RV) failure and death.

The right heart catheterization (RHC) examination is the gold standard for the diagnosis of PH. But because it is an invasive operation, RHC can lead to adverse events related to venous access complications (such as hematoma and pneumothorax), and can also lead to arrhythmia, vagal nerve reaction, or pulmonary vascular reactivity test of hypotension; thus, coupled with the facilities of basic units, this can limit the application of RHC in the diagnosis of PH to a certain extent, and also lead to a delay of PH treatment, reducing the prognosis of patients (5). Due to the non-specificity of its symptoms, PH is often diagnosed late. Given the availability of therapies for specific forms of PH, there is increasing interest in better patient phenotyping and improving diagnostic rates with imaging (6). In recent years, as a non-invasive examination method, computed tomography (CT) has played an important role in the diagnosis of PH. Many studies have explored the diagnostic value of CT for PH in various aspects (7). The ratio of the main pulmonary artery to ascending aorta is a tool for assessing the dilation of pulmonary arterial segments because PH causes the selective enlargement of the main pulmonary artery and its major branches, but not of the ascending aorta. Corson et al. (8) also indicated the determination of pulmonary artery diameter and ascending aorta diameter to calculate the MPAD/AD ratio with MPAD/AD >1 as PH. This study conducted a meta-analysis on the relevant literature published in recent years to evaluate the value of CT in the diagnosis of PH and provide a reliable diagnostic method for its early diagnosis.

## Materials and methods

### Literature search

Searched databases included PubMed, Embase, Scopus, and Web of Science up to May 2022. The PubMed retrieval formula is [“Tomography, X-Ray Computed” (Mesh)] OR [Tomography, X-Ray Computerized (Title/Abstract)] OR [Tomography, X-Ray Computerized (Title/Abstract)] OR [Computed X-Ray Tomography (Title/Abstract)] OR [X-Ray Computer Assisted Tomography (Title/Abstract)] OR [X-Ray Computer Assisted Tomography (Title/Abstract)] OR [Tomography, X-Ray Computer Assisted (Title/Abstract)] OR [Tomography, X-Ray Computer Assisted (Title/Abstract)] OR [Computerized Tomography, X-Ray (Title/Abstract)] OR [Computerized Tomography, X-Ray (Title/Abstract)] OR [X-Ray Computerized Tomography (Title/Abstract)] OR [CT X-Ray (Title/Abstract)] OR [CT X-Rays (Title/Abstract)] OR [X-Ray, CT (Title/Abstract)] OR [X-Rays, CT (Title/Abstract)] OR [Tomodensitometry (Title/Abstract)] OR [Tomography, X-Ray Computed (Title/Abstract)] OR [X-Ray Tomography, Computed (Title/Abstract)] OR [X-Ray Tomography, Computed (Title/Abstract)] OR [Computed X-Ray Tomography (Title/Abstract)] OR [Tomographies, Computed X-Ray (Title/Abstract)] OR [Tomography, Computed X-Ray (Title/Abstract)] OR [Tomography, X-Ray Computed (Title/Abstract)] OR [Computed Tomography, X-Ray (Title/Abstract)] OR [X-Ray Computed Tomography (Title/Abstract)] OR [CAT Scan, X-Ray (Title/Abstract)] OR [CAT Scan, X-Ray (Title/Abstract)] OR [CAT Scans, X-Ray (Title/Abstract)] OR [Scan, X-Ray CAT (Title/Abstract)] OR [Scans, X-Ray CAT (Title/Abstract)] OR [X-Ray CAT Scan (Title/Abstract)] OR [X-Ray CAT Scans (Title/Abstract)] OR [Tomography, Transmission Computed (Title/Abstract)] OR [Computed Tomography, Transmission (Title/Abstract)] OR [Transmission Computed Tomography (Title/Abstract)] OR [CT Scan, X-Ray (Title/Abstract)] OR [CT Scan, X-Ray (Title/Abstract)] OR [CT Scans, X-Ray (Title/Abstract)] OR [Scan, X-Ray CT (Title/Abstract)] OR [Scans, X-Ray CT (Title/Abstract)] OR [X-Ray CT Scan (Title/Abstract)] OR [X-Ray CT Scans (Title/Abstract)] OR [Computed Tomography, X-Ray (Title/Abstract)] OR [Computed Tomography, X-Ray (Title/Abstract)] OR [X-Ray Computerized Tomography (Title/Abstract)] OR [Cine-CT (Title/Abstract)] OR [Cine CT (Title/Abstract)] OR [Electron Beam Computed Tomography (Title/Abstract)] OR [Electron Beam Tomography (Title/Abstract)] OR [Beam Tomography, Electron (Title/Abstract)] OR [Tomography, Electron Beam (Title/Abstract)] OR [Tomography, X-Ray Computerized Axial (Title/Abstract)] OR [Topography, X-Ray Computerized Axial (Title/Abstract)] OR [X-Ray Computerized Axial Tomography (Title/Abstract)] OR [X-Ray Computerized Axial Tomography (Title/Abstract)] AND [“Pulmonary Arterial Hypertension” (Mesh)] OR [Hypertension, Pulmonary (Title/Abstract)]. To avoid literature omission, the references of the searched documents were searched manually.

### Inclusion criteria

(1) Research subjects: patients with PH, the gold standard is RHC examination mPAP ≥25 mmHg in resting state. (2) Research type: non-animal experiments. (3) Intervention measures: accept RHC and CT examination. (4) Research results: the literature can directly give true positive (TP), false positive (FP), true negative (TN), and false negative (FN) through calculation. (5) The language is English.

### Exclusion criteria

(1) Literature for review and conference reports; (2) data duplication; (3) does not include test indicators to be evaluated; and (4) Grade C quality standard.

### Literature screening and data extraction

The titles, abstracts and texts were independently examined by two researchers (HC and ZZ) to select eligible studies for full-text level review. The same two researchers independently extracted data from eligible original studies. The main extracted data were author, year, country, study type, sample size, specificity, sensitivity, etc. Disagreements regarding data extraction and literature screening were resolved through discussion with the third researcher (RC).

### Literature quality evaluation

For the final included articles, the two researchers evaluated the risk of literature bias according to the QUADAS-2 tool (9) in the aspects of case selection, trial, gold evaluation, gold standard, process and progress, and the literature was finally determined as Grades A, B, and C. Inconsistent evaluation results were discussed with the third researcher.

### Statistical analysis

Deek’s funnel plots were made using Stata 12.0 software, and publication bias was analyzed across the studies. *P* > 0.05 meant no obvious publication bias. Spearman’s correlation coefficient was calculated using Meta-DiSc 1.40 software to analyze whether there was a threshold effect between studies. Stata 12.0 was used to analyze the sensitivity and specificity of heterogeneity among the various studies. If *P* > 0.05 and *I*^2^ < 50%, it was considered that there was no heterogeneity between the studies, so a fixed effects model was used; if *P* < 0.05 and *I*^2^ > 50%, it was considered that there was heterogeneity among the studies, and meta-regression and sensitivity analysis were performed to explore the source of heterogeneity. If the source of heterogeneity could not be excluded, a random effects model was used. The comprehensive summary receiver operating characteristic (SROC) curve was mapped and the area under the curve (AUC) was calculated.

## Results

### Literature search results

**FIGURE 1:**
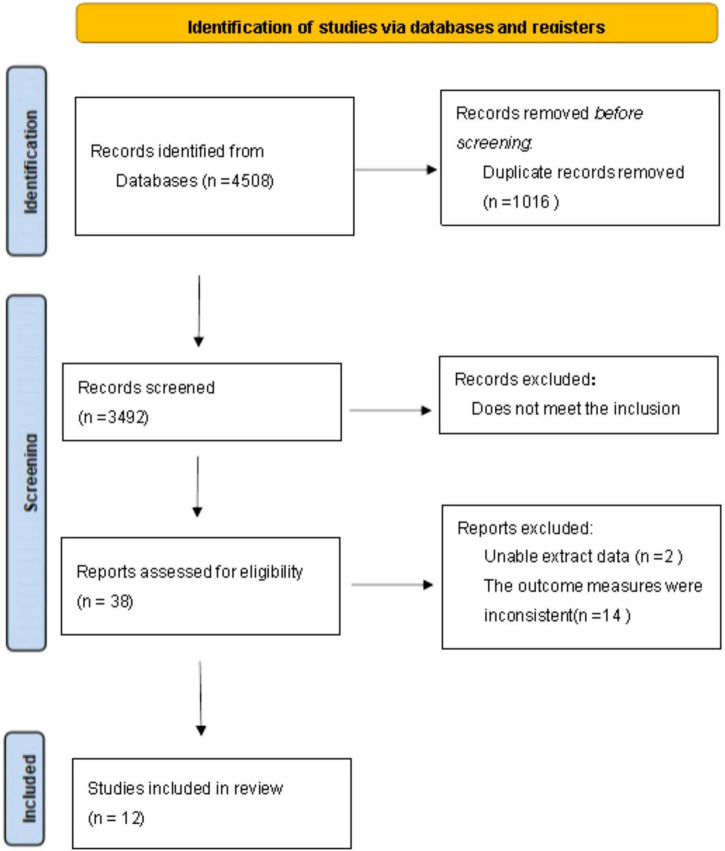
Flow chart of literature screening.

After searching as described above, 4,508 documents were retrieved, of which 4,470 were excluded. After screening by inclusion and exclusion criteria, 38 remained. Of these, 14 did not use pulmonary artery diameter/liter aortic diameter >1 or 1 as the diagnostic criteria for PH, and 2 did not directly or indirectly calculate the four-grid table data. Therefore, twelve articles were finally included. The screening process is shown in Figure 1.

### Data extraction and literature quality evaluation

**FIGURE 2:**
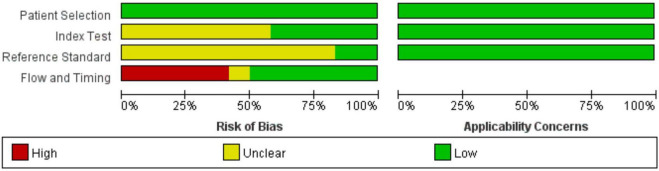
QUADAS-2 quality evaluation of literature.

**FIGURE 3:**
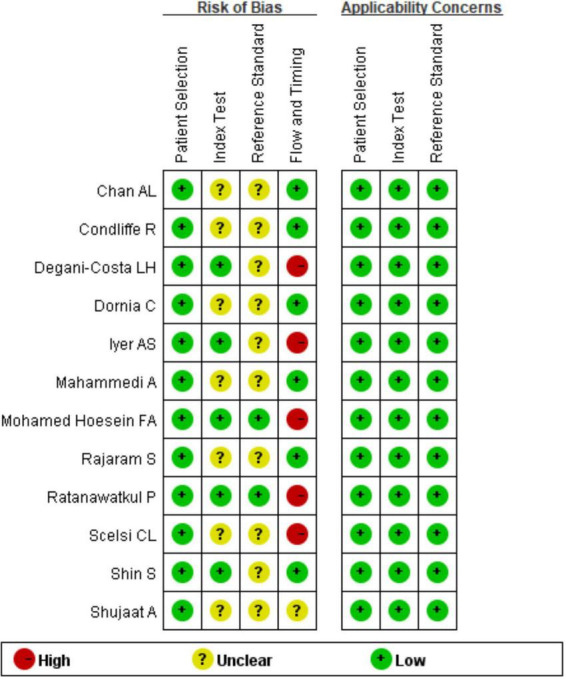
QUADAS-2 quality evaluation of literature.

The 10 included articles (10–21) were published from 2011 to 2020, with a total of 1,959 subjects. The basic characteristics of each articles are shown in Table 1. All 10 studies included were retrospective, from the UK, USA, Netherland, and Brazil, and five (13, 15, 16, 19, 21) had underlying diseases such as systemic sclerosis, connective tissue disease and COPD respectively. According to the QUADAS-2 standard, all articles were included in case selection without case control and inappropriate exclusion. In the test and gold standard, Iyer et al. (15), Shin et al. (16), Degani-Costa et al. (18), and Mohamed Hoesein et al. (20) showed that the results were unknown, Shujaat et al. (10) did not mention the interval between study CT and RHC, and the remaining studies were controlled within half a year. All twelve articles were Grade B or above (see Figures 2, 3).

**TABLE 1:** Basic characteristics of the included literature.

References	Age (year)	Background disease	Time interval (day)	Study type	Country	Sample	TP	FP	FN	TN
Shujaat et al. (10)	54.3	No	/	Retrospective	America	87	49	7	16	15
Chan et al. (11)	61.5	No	16	Retrospective	America	101	46	10	7	38
Dornia et al. (12)	58	No	90	Retrospective	Britain	172	72	4	42	54
Rajaram et al. (13)	62	Connective tissue disease	2	Retrospective	America	77	30	6	25	16
Mahammedi et al. (14)	63.5	No	90	Retrospective	America	400	211	24	87	78
Iyer et al. (15)	56.25	COPD	120	Retrospective	America	60	16	6	6	32
Shin et al. (16)	59	COPD	90	Retrospective	America	65	19	2	19	25
Scelsi et al. (17)	64	No	120	Retrospective	America	186	72	11	55	48
Degani-Costa et al. (18)	58.22	No	180	Retrospective	Brazil	32	11	0	4	17
Condliffe et al. (19)	66	Systemic sclerosis	90	Retrospective	Britain	67	38	2	10	17
Mohamed Hoesein et al. (20)	70.1	No	60	Retrospective	America	620	199	37	176	208
Ratanawatkul et al. (21)	55.1	COPD	180	Retrospective	Netherland	92	15	9	15	53

TP, true positive; FP, false positive; TN, true negative; FN, false negative.

### Analysis of statistical results

#### Meta-analysis results

**FIGURE 4:**
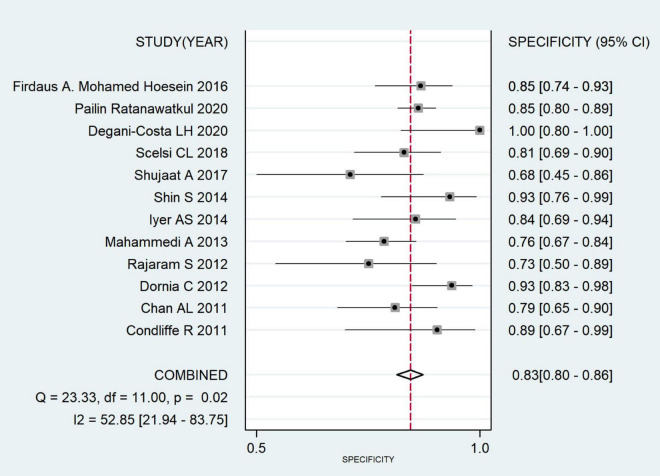
Forest plot of CT combined sensitivity of pulmonary hypertension.

**FIGURE 5:**
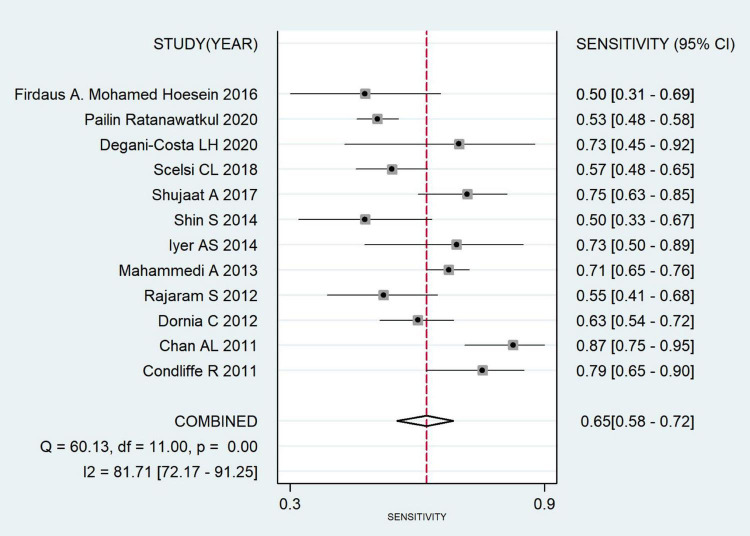
Forest plot of pulmonary hypertension diagnosed with specificity by CT.

**FIGURE 6:**
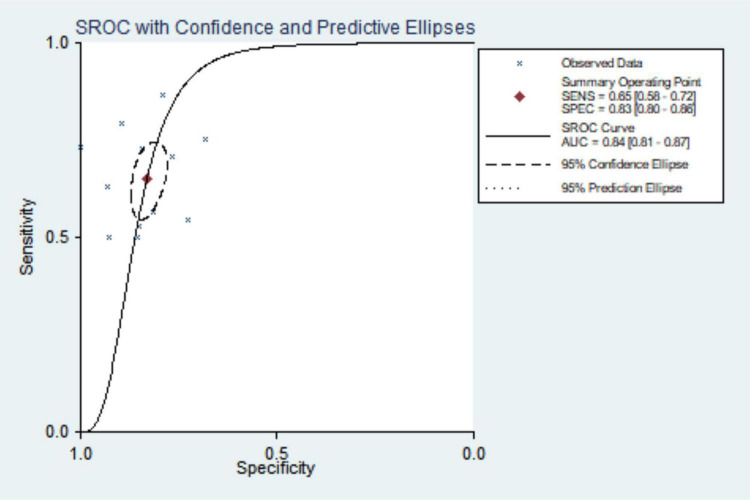
Summary receiver operating characteristic curve for CT diagnosis of pulmonary hypertension.

The combined sensitivity was 0.652 (95% CI: 0.579, 0.719), combined specificity was 0.830 (95% CI: 0.796, 0.880), positive likelihood ratio was 3.837 (95% CI: 3.215, 4.579), negative likelihood ratio was 0.419 (95% CI: 0.346, 0.507), diagnostic odds ratio was 9.157 (95% CI: 6.748, 12.427) and area under the SROC curve was 0.84 (95% CI: 0.81, 0.87). The specificity and sensitivity forest map and SROC curves are shown in Figures 4–6.

#### Heterogeneity test

Pooled heterogeneity analysis yielded *I^2^* = 89.41%, *P* < 0.001. Large heterogeneity existed in the included original literature, so a random effects model was selected to incorporate the statistics. The Spearman correlation coefficient was calculated as 0.224 (*P* = 0.484), showing no threshold effect. Sources of heterogeneity were explored by subgroup analysis.

#### Subgroup analysis

Subgroup analysis was performed by country, etiology typing, time interval, sample size, and year, and the summary results are shown in Table 2. The results of the subgroup analysis suggested that countries and time intervals may be sources of heterogeneity.

**TABLE 2:** Diagnostic accuracy summary of subgroup analysis.

	SEN (95% CI)	SPE (95% CI)	AUC (95% CI)	Heterogeneity (%)
**Country**				
America	0.650 (0.559, 0.732)	0.809 (0.764, 0.848)	0.82 (0.79, 0.85)	91.17
Other	0.666 (0.543, 0.769)	0.919 (0.836, 0.962)	0.90 (0.87, 0.92)	0.0
**Causal classification**				
Secondary cause	0.620 (0.513, 0.717)	0.822 (0.754, 0.874)	0.82 (0.79, 0.85)	65.92
All types	0.680 (0.577, 0.768)	0.837 (0.793, 0.873)	0.85 (0.82, 0.88)	81.88
**Time interval**				
>90	0.557 (0.489, 0.623)	0.867 (0.806, 0.911)	0.81 (0.77, 0.84)	0.0
≦90	0.685 (0.582, 0.772)	0.825 (0.714, 0.899)	0.84 (0.81, 0.87)	87.30
**Sample**				
<100	0.646 (0.561, 0.724)	0.823 (0.753, 0.877)	0.82 (0.78, 0.85)	67.91
>100	0.663 (0.533, 0.773)	0.843 (0.799, 0.878)	0.85 (0.82, 0.88)	75.02
**Year**				
Before 2015	0.689 (0.592, 0.771)	0.843 (0.774, 0.893)	0.85 (0.82, 0.88)	76.81
After 2015	0.594 (0.501, 0.680)	0.827 (0.767, 0.875)	0.80 (0.77, 0.84)	53.22

AUC, area under the curve; SEN, sensitivity; SPE, specificity.

#### Sensitivity analysis

**FIGURE 7:**
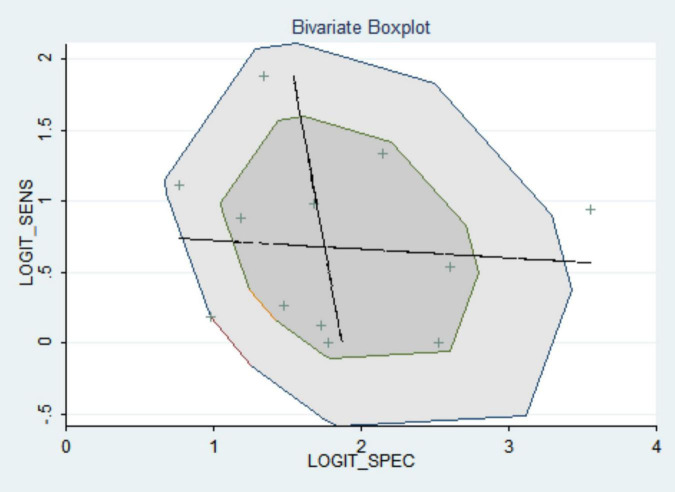
Bivariate boxplot.

The included articles were excluded one by one, and the sensitivity and specificity of the observed merger did not change greatly, which shows the stability and credibility of the merger results of this meta-analysis (see Figure 7).

#### Publication bias

**FIGURE 8:**
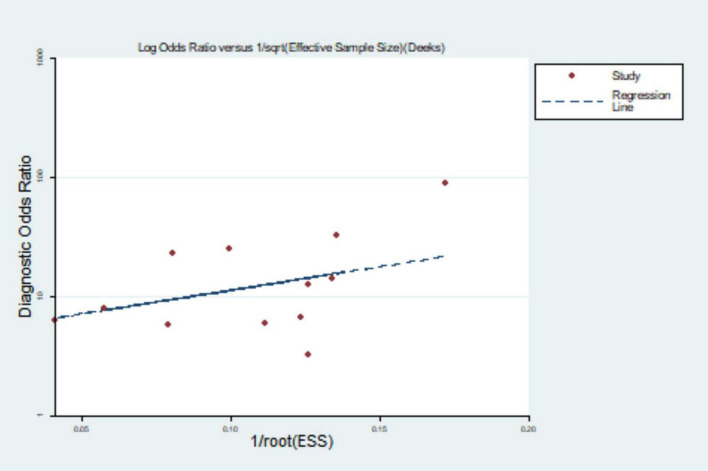
Deek’s funnel diagram.

Mapping the Deek’s funnel plot detected publication bias of *P* = 0.102, indicating that there was no publication bias (see Figure 8).

## Discussion

Pulmonary hypertension is a rare distal pulmonary artery disease characterized by vasoconstriction, vascular proliferation, obstructive remodeling of the pulmonary vascular walls, inflammation and thrombosis, leading to a gradual increase in pulmonary vascular resistance and ultimately RV failure and death (22). Chronic inflammation and damaged vascular endothelial cells are involved in the process of vascular remodeling, vascular smooth muscle cell hyperplasia and vascular thickening, leading to increased pulmonary artery pressure. Chest CT scan can measure increases in pulmonary artery trunk diameter to diagnose PH (23). With the development of CT and computer technology, chest CT scan by X-ray radiation can provide relatively good image quality, reflect the subtle structure of the lung, greatly enhance lung function ability, benefit the discovery of lung anatomy, and clearly show lung interstitium, parenchyma, mediastinal, and heart function changes, thereby assisting in the clinical cause of PH (24).

Currently, there are various criteria for CT the diagnosis of PH, including pulmonary artery diameter/ascending aortic diameter, contrast flow rate, contrast peak time, RV free wall/LV free wall, RV cavity diameter/LV cavity diameter, etc. (25, 26). Most studies determine the pulmonary artery diameter and ascending aorta diameter, and calculate the MPAD/AD ratio with MPAD/AD >1 as PH. Pulmonary artery diameter can directly reflect the vascular dilation and blood vessel thickening conditions, so measuring MPAD can more effectively reflect pulmonary artery pressure. However, pulmonary artery vascular dilation is affected by many factors such as age, body surface area, vascular compliance, etc., the MPAD value alone can reflect the pulmonary artery pressure of different individuals, and the MPAD/AD ratio can eliminate individual differences to a certain extent (27).

A total of ten articles were included in this study, all at Grade B or above after evaluation. They all used pulmonary artery diameter/ascending aortic diameter >1 or 1 as the diagnostic criteria for PH. According to the meta-analysis, the pooled sensitivity and specificity were 0.652 (95% CI: 0.579, 0.719) and 0.830 (95% CI: 0.796, 0.880), the area under the SROC curve was 0.84 (95% CI: 0.81, 0.87), and the sensitivity was somewhat insufficient, but the specificity was good, indicating that chest CT using MPAD/AD ratio to diagnose PH has good predictive value. There was great heterogeneity in the original literature included in this study, and the results of subgroup analysis suggest that countries and time interval may be the source of heterogeneity. The robustness of the analysis results was verified by sensitivity analysis. The Deek’s funnel plot shows the inclusion of the articles without publication bias.

The limitations of this meta-analysis are as follows: (1) the included literature is only in English without studies in other languages; (2) the heterogeneity is large, still need to standardize, large sample, multi-center clinical research further confirmed; (3) the outcome index is only pulmonary artery diameter/liter aortic diameter without other criteria for the CT diagnosis of PH.

## Conclusion

In conclusion, pulmonary artery diameter/liter aortic diameter >1 or 1 can be used as a method for diagnosing PH by CT, which is easy to operate and has important applications. CT can provide a reliable diagnostic method for the early diagnosis of PH.

## Data availability statement

The raw data supporting the conclusions of this article will be made available by the authors, without undue reservation.

## Ethics statement

Ethical review and approval and written informed consent from the patients/participants was not required for this study in accordance with the local legislation and institutional requirements.

## Author contributions

RC, HL, HC, CH, and NZ were the guarantor of the manuscript and took responsibility for the content of this manuscript. RC, CH, and NZ contributed to the design of the study. ZD, ZFH, and ZZ were involved in the data analysis. MJ, XW, WG, ZJH, and JL contributed to the acquisition of primary data. HL and RC wrote the initial draft of the manuscript. HC, NZ, and CH contributed significantly to the revision of the manuscript. All authors read and approved the final manuscript.
